# Mechanisms of Isoform Specific Rap2 Signaling during Enterocytic Brush Border Formation

**DOI:** 10.1371/journal.pone.0106687

**Published:** 2014-09-09

**Authors:** Lucas J. M. Bruurs, Johannes L. Bos

**Affiliations:** Molecular Cancer Research and Cancer Genomics Netherlands, Center for Molecular Medicine, University Medical Center Utrecht, Utrecht, The Netherlands; Institute of Biology Valrose, France

## Abstract

Brush border formation during polarity establishment of intestinal epithelial cells is uniquely governed by the Rap2A GTPase, despite expression of the other highly similar Rap2 isoforms (Rap2B and Rap2C). We investigated the mechanisms of this remarkable specificity and found that Rap2C is spatially segregated from Rap2A signaling as it is not enriched at the apical membrane after polarization. In contrast, both Rap2A and Rap2B are similarly located at Rab11 positive apical recycling endosomes and inside the brush border. However, although Rap2B localizes similarly it is not equally activated as Rap2A during brush border formation. We reveal that the C-terminal hypervariable region allows selective activation of Rap2A, yet this selectivity does not originate from the known differential lipid modifications of this region. In conclusion, we demonstrate that Rap2 specificity during brush border formation is determined by two distinct mechanisms involving segregated localization and selective activation.

## Introduction

GTPases of the Rap family function as molecular switches that relay signals under strict control of spatial and temporal cues. Three Rap2 proteins exist (Rap2A, Rap2B and Rap2C) that are over 90% identical in protein sequence. As a result of this sequence similarity none of the currently known Rap2GEFs, GAPs and effectors have preferred activity towards a single isoform *in vitro* [1]. Nevertheless, isoform specific signaling routes have been described raising the question how these highly similar GTPases can achieve signaling selectivity *in vivo* [2]–[4].

**Figure 1:**
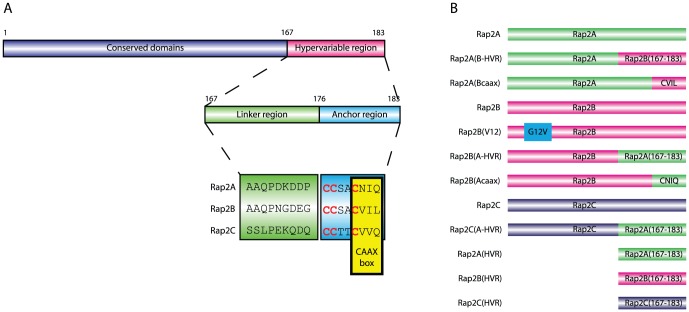
General structure of the Rap2 proteins and constructs used in this study. A: Domain structure of Rap2 isoforms. Proteins differ most extensively in the C-terminal hypervariable region (HVR), which comprises a linker and anchor region. Cysteine residues in the anchor region (highlighted in red) are posttranslationally subjected to CAAX box isoprenylation and dynamic cysteine palmitoylation. B: Schematic structure of various Rap2 constructs used in this study.

In the context of the closely related K-, H- and N-Ras proteins, isoform selectivity originates from the C-terminal hypervariable region (HVR) [5], [6]. The HVR constitutes the major localization signal for GTPases as it is the site for both irreversible CAAX prenylation and dynamic cysteine palmitoylation. These lipid modifications allow for membrane association that in combination with the presence of a polybasic region or additional sorting signals in the HVR determine the localization of the Ras GTPases [7], [8]. However, in comparison with the HVRs of the Ras isoforms, the Rap2 isoforms have very similar HVR compositions. Nevertheless, the Rap2 HVRs differ in amino acid sequence and in CAAX-box modification: whereas Rap2A and Rap2C are farnesylated, the Rap2B isoform is modified with a geranylgeranyl moiety [9], [10] (figure 1a).

In the intestinal epithelial Ls174T-W4 cell line, single cells can polarize upon doxycycline-induced expression of STRAD and the subsequent cytosolic stabilization and activation of LKB1 [11]. Previously, we identified a pathway in which the small GTPase Rap2A regulates apical brush border formation downstream of LKB1 [2]. Interestingly, only knockdown of Rap2A prevents brush border formation during Ls174T-W4 polarization, whereas knockdown of the other Rap2 isoforms present in this cell line has no effect. Notably, neither the upstream activator (PDZGEF) nor the downstream effector (TNIK) of Rap2A in this pathway have specificity towards any of the Rap2 isoforms [1], [9]. Therefore, we set out to investigate the mechanisms via which isoform specific Rap2 signaling is achieved during brush border formation. Using an imaging approach in combination with mutant analysis we find that Rap2C is spatially isolated from Rap2A signaling. In contrast, Rap2B, although identically localized, is not similarly activated as Rap2A during brush border formation. We identify the C-terminal hypervariable region to be responsible for differential activation between Rap2A and Rap2B. Therefore, we conclude that the hypervariable region of Rap2 can regulate protein activity independent of its effects on localization and thereby allows for isoform specific Rap2 signaling during brush border formation.

## Methods

### Cell culture and constructs

Ls174T-W4 cells [11] were cultured in RPMI1640 (Lonza) supplemented with 10% tetracycline-free FBS (Lonza) and antibiotics. For transient transfection, cells, while in suspension, were transfected with X-tremeGene 9 (Roche) according to the manufacturer's protocol. To induce polarization, cells were trypsinized and seeded in doxycycline-containing medium (1 µg/ml) for at least 16 h.

N-terminally GFP-, CFP- and V5- tagged Rap2 constructs were cloned using Gateway recombination (Invitrogen) in a pcDNA3 backbone. HVR- and CAAX-mutants were generated by site-directed mutagenesis and the isolated HVR constructs were generated by directly ligating annealed oligo's into pEGFP-c2 using the EcoRI and SalI restriction sites.

### Lentiviral knockdown

Ls174T-W4 cells were infected for two successive days with lentiviral shRNA constructs (Mission library, Sigma). Three days after the first round of infection, infected cells were selected with puromycin (10 µg/ml) for three days. For stable knockdown, four shRNAs targeting the Rap2A mRNA were pooled (Targeting sequences shRNA #1: 5′-CGGCACCTTCATCGAGAAATA-3′, shRNA #2: 5′-CCTTTATGGAAACTTCCGCTA-3′, shRNA #3: 5′-GACGAACTCTTTGCAGAAATT-3′, shRNA #4: 5′-GTATGAGAAAGTGCCAGTCAT-3′), whereas for rescue experiments with Rap2A(B-HVR) and CAAX mutants a single Rap2A shRNA was used, targeting the sequence encoding the Rap2A HVR. (Targeting sequence shRNA #5: 5′-GTTCTGCATGTAACATACAAT-3′) shRNAs were validated to have no compensatory effects on Rap2B and Rap2C mRNA levels. (figure S1 in File S1 and [2]).

### Life cell imaging

Two days after transfection, cells were stimulated with doxycycline and seeded on glass bottom dishes (WillCo Wells). After at least 16 h, cells were imaged in Hepes-buffered (pH 7.4) Leibovitz's L-15 medium (Invitrogen) at 37°C on an Axioskop2 LSM510 confocal microscope (Zeiss). Brush border formation was quantified in three independent experiments for a total of at least 150 cells and averages before normalization were compared with control transfected W4 cells using paired samples t-test.

### Rap activity assay

V5-Rap2 constructs were transfected in W4 cells and cells were stimulated with doxycycline for various time points. Cells were gently scraped in cold lysis buffer [1% Nonidet P-40 substitute; 10% glycerol; 50 mM Tris-HCl pH 7.4; 2.0 mM MgCl_2_; 200 mM NaCl; protease and phosphatase inhibitors] and cleared by centrifugation at 4°C. Protein concentration of the cleared lysates was quantified using the BCA protein assay (Thermo) and equal amounts of protein were subjected to precipitation with glutathione-agarose beads coupled with the GST fusion protein of the Ras-binding domain of RalGDS (GST-RalGDS-RBD) [12]. Beads were washed, eluted in Laemmli sample buffer and resolved by SDS PAGE. Western blots were probed with anti-panRap2 (BD Biosciences), anti-V5 (Invitrogen), anti-αTubulin (Calbiochem) and anti-FLAG (M2) (Sigma).

Blots from three independent experiments were quantified using ImageJ software. For this the ratio between band intensities of the V5 signal in pulldown and total lysates was determined and expressed relative to V5-Rap2A without doxycycline stimulation (Or V5-Rap2B in figure S4 in File S1). Quantifications show the average ratios in three experiments with error bars representing standard deviations.

## Results

### Rap2C is differentially localized, whereas Rap2B localizes similarly as Rap2A in polarized Ls174T-W4 cells

**Figure 2:**
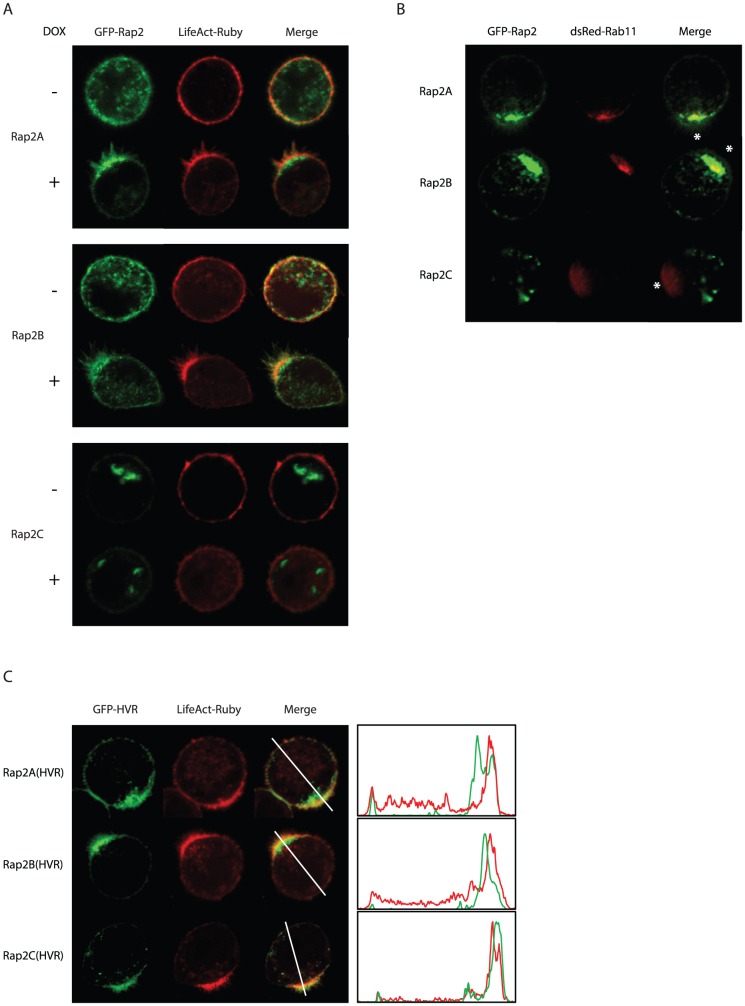
Localization of Rap2 isoforms in polarized Ls174T-W4 cells. A: GFP-tagged Rap2 isoform were cotransfected with the actin marker Lifeact-Ruby in W4 cells and imaged in unpolarized and polarized (i.e. doxycycline-stimulated) cells. B: GFP-Rap2 localization compared with the recycling endosomal marker dsRed-Rab11 in polarized W4 cells. Asterisks indicate the apical aspect as judged by polarized Rab11 distribution. C: Localization of the isolated hypervariable regions of the Rap2 isoforms. Profile plots show normalized fluorescence intensities over the indicated line scans. DOX: doxycyline.

Since Rap GTPases are known to signal from distinct spatial pools, we assessed localization of the different Rap2 proteins in Ls174T-W4 cells [13]. After doxycycline-induced polarization, we observe enrichment of Rap2A at the apical brush border as judged by colocalization with the actin cytoskeleton marker Lifeact-Ruby [14] (figure 2A). Interestingly, Rap2B shows a similar localization upon polarization as Rap2A: both isoforms are predominantly located in the brush border membrane and in cytosolic vesicles subjacent to the brush border. These vesicles colocalize with the apical recycling endosome marker Rab11 (figure 2B). Recycling endosomes are pivotal for the establishment of apical membranes by sorting multiple signaling proteins to the apical membrane [15], [16]. Thus, we identify two distinct pools of Rap2 at the apical aspect of polarized Ls174T-W4 cells: a pool residing on Rab11 positive endosomes *en route* to the apical plasma membrane and a second pool residing at the plasma membrane in the brush border.

Whereas Rap2A and Rap2B become enriched at the apical aspect upon polarization, Rap2C remains localized in vesicles that do not colocalize with dsRed-Rab11 (figure 2B). Therefore, we conclude that for Rap2C, but not Rap2B, differential localization most likely underlies the failure to function in brush border formation.

### The hypervariable region of Rap2A and Rap2B allows for selective localization in polarized Ls174T-W4 cells

To get further insight in why Rap2C is differentially and Rap2B is similarly localized as Rap2A we assessed the localization of the isolated C-terminal HVRs as this region is most divergent between Rap2 isoforms and is known to regulate localization of other GTPases [6], [8].

After Ls174T-W4 cell polarization, both GFP-Rap2A(HVR) and GFP-Rap2B(HVR) localize on recycling endosomes and at the brush border membrane, reflecting distribution of the full length proteins (figure 2C). In contrast, GFP-Rap2C(HVR) only localizes at the brush border and not on recycling endosomes. Therefore, we conclude that the HVR of Rap2A and Rap2B contains the dominant localization signals for these proteins, whereas for Rap2C localization of the isolated HVR (plasma membrane bound) does not reflect localization of the full length protein (vesicular), indicating that for Rap2C additional sorting signals overrule HVR-imposed localization. Furthermore, differences in the HVR of Rap2A and Rap2B compared to the HVR of Rap2C imposes specific localization at the recycling endosomes for Rap2A and Rap2B. Taken together, this demonstrates that the HVR of Rap2A and Rap2B selectively imposes localization of the full length proteins in polarized W4 cells.

### Rap2A and Rap2B are differentially activated during brush border formation

**Figure 3:**
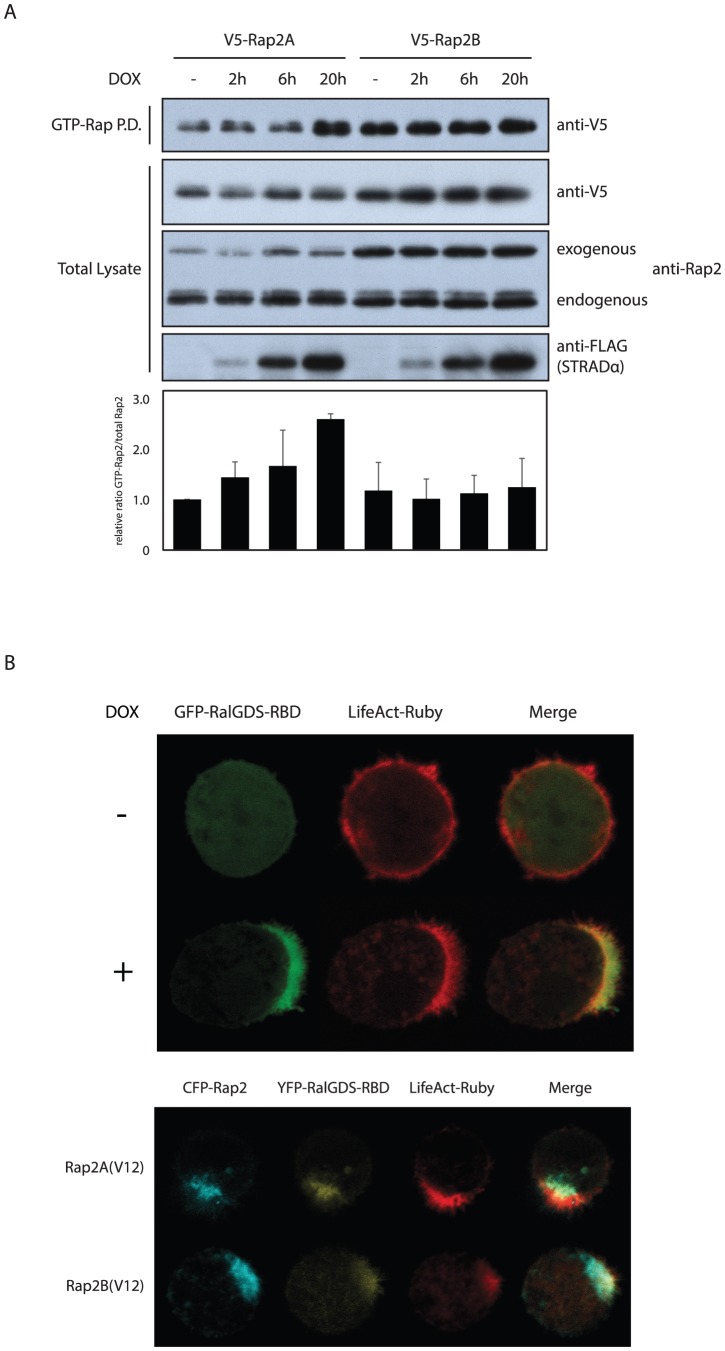
Rap2A and Rap2B are differentially activated during Ls174T-W4 cell polarization. A: Pulldown of GTP-bound V5-Rap2A and V5-Rap2B from W4 cells stimulated with doxycycline for various durations. Total lysates were probed for V5, panRap2 and FLAG. FLAG-STRADα levels were assessed to demonstrate differential induction over the different time points. Results from three independent experiments were averaged and expressed as the ratio of GTP-Rap2 vs. total Rap2 signal relative to unstimulated V5-Rap2A. Error bars represent standard deviations. B: Top panel: Distribution of GTP-bound Rap proteins in unpolarized and polarized W4 cells as visualized by localization of the Ras-binding domain (RBD) of RalGDS. Bottom panel: localization of YFP-RalGDS-RBD in polarized W4 cells expressing constitutively activated CFP-Rap2A(V12) or CFP-Rap2B(V12). GTP-Rap P.D.: GTP-bound Rap pulldown, DOX: doxycyline.

Since localization does not explain specificity of Rap2A over Rap2B we questioned whether the activity of Rap2B is similarly induced as Rap2A during brush border formation. To assess this, we transfected V5-tagged Rap2A and Rap2B and subsequently used the Ras-binding domain of RalGDS (RalGDS-RBD) to specifically pulldown GTP-loaded Rap proteins [12]. After doxycycline treatment to induce brush border formation, an increase in GTP-bound Rap2A is observed whereas no induction in Rap2B (and Rap2C) activity is detected (figure 3A and figure S2 in File S1). This indicates that Rap2B activity is not similarly controlled as Rap2A during brush border formation and suggests that for these proteins isoform specificity results from differential activation.

In order to visualize the site of Rap activation we expressed GFP-RalGDS-RBD in W4 cells and found that after polarization it is exclusively recruited to the brush border and not to the recycling endosomes (figure 3B). In contrast, when either constitutively activated CFP-Rap2A(V12) or CFP-Rap2B(V12) were present, YFP-RalGDS-RBD was able to localize to the recycling endosomes (figure 3B). These results indicate that the pool of Rap2A and Rap2B on the recycling endosomes are maintained in an inactive conformation and that Rap2A is selectively activated upon disassembly of the recycling endosomes at the apical membrane.

To further test the hypothesis that Rap2A and Rap2B differ in their activation state at the brush border, we scored brush formation in W4 cells stably depleted of endogenous Rap2A in which various Rap2 mutants were added back (figure 1B).

**Figure 4:**
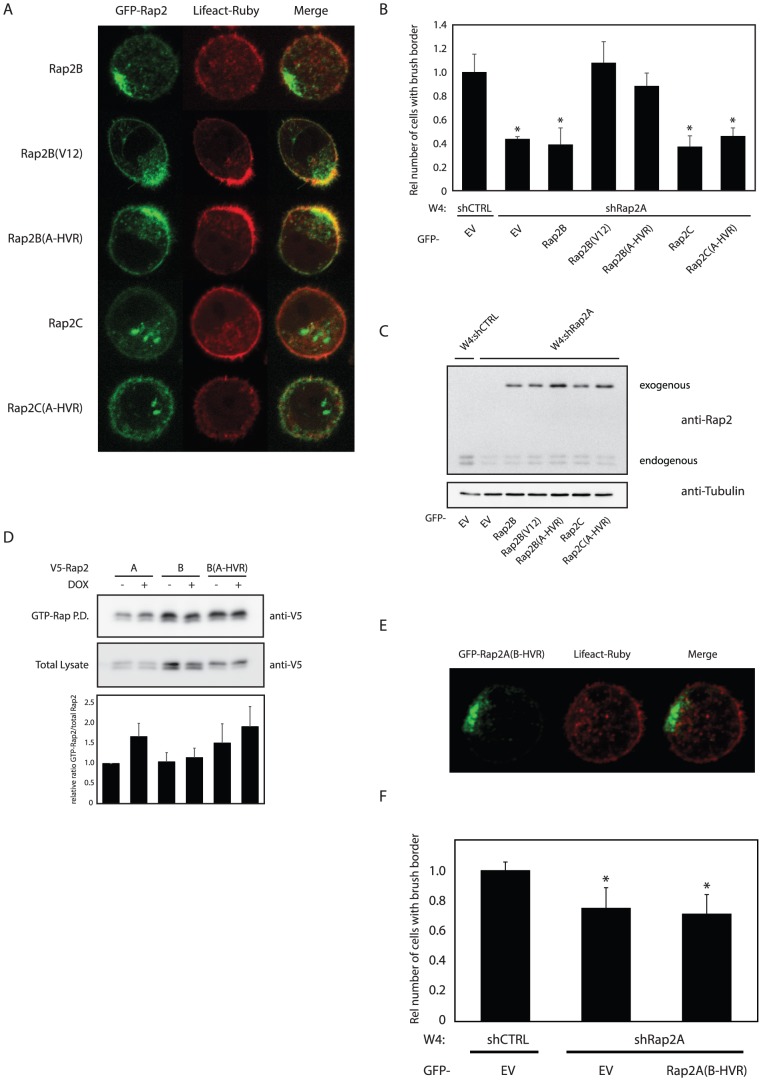
Constitutively activated Rap2B(V12) or a chimeric Rap2B(A-HVR) mutant can rescue brush border formation in Rap2A depleted Ls174T-W4 cells. A: Images of Rap2A depleted polarized W4 cells expressing various GFP-tagged Rap2 constructs and LifeAct-Ruby. B: Quantification of brush border formation in GFP-positive cells in three independent experiments. (total counts ∼150 cells per condition). *p<0,05 using paired samples t-test. C: Western blot of lysates from a rescue experiment probed with anti-Rap2 antibody and anti-αTubulin as a loading control. D: GTP-Rap pulldown from unpolarized and polarized W4 cells expressing V5-Rap2A, V5-Rap2B or V5-Rap2B(A-HVR). Results from three independent experiments were quantified and averages were expressed as ratio of GTP-Rap2 vs. total Rap2 relative to unstimulated Rap2A. E: Image of Rap2A-depleted W4 cell expressing GFP-Rap2A(B-HVR) and LifeAct-Ruby. F: Quantification of brush border formation in Rap2A depleted W4 cells in which GFP or GFP-Rap2A(B-HVR) was introduced. (Total counts ∼300 cells per condition). *p<0,05 using paired samples t-test. shCTRL: non-targeting control short hairpin, EV: empty vector, DOX: doxycycline, GTP-Rap P.D.: GTP-bound Rap pulldown.

Importantly, overexpression of wild type GFP-Rap2B and GFP-Rap2C cannot overcome the brush border formation defect imposed by Rap2A depletion (figure 4A & B). This again emphasizes that Rap2A is the dominant isoform during brush border formation and also demonstrates that isoform selectivity is maintained under overexpression conditions. In contrast, when overexpressing a constitutively active Rap2B(V12) mutant, brush border formation was restored in Rap2A depleted W4 cells. The Rap2B(V12) mutant is similarly localized as wild type Rap2B, yet it is highly GTP-loaded because of reduced sensitivity towards GAP-mediated inactivation (figure 4A & B). No rescue of brush border formation was observed when Rap2C(V12) was expressed in Rap2A depleted W4 cells (Figure S3 in File S1).

Together with the failure of Rap2B to become activated after polarization, these findings suggest that although properly localized, Rap2B is not similarly activated as Rap2A and as a result of this does not contribute to brush border formation.

### Differences in the hypervariable region determine signaling specificity between Rap2A and Rap2B

Next, we addressed whether the hypervariable region of Rap2A allows for selective Rap2A activation at the brush border. For this we generated chimeric Rap2 proteins in which the hypervariable region of Rap2B (and Rap2C) was replaced by the HVR of Rap2A (figure 1B). When expressed in W4 cells depleted of endogenous Rap2A, GFP-Rap2B(A-HVR) was able to restore brush border formation (figure 4A & B). In agreement with this, we find that V5-Rap2B(A-HVR) is more GTP-loaded in polarized W4 cells compared to wild type V5-Rap2B (figure 4D).

Furthermore, a chimeric Rap2A(B-HVR) mutant loses the ability to induce brush border formation in Rap2A depleted W4 cells (figure 4E & F) and is less active in polarized W4 cells compared to Rap2B(A-HVR) (figure S4 in File S1). Therefore, we conclude that differences in signaling by Rap2A and Rap2B can be attributed solely to differences in the hypervariable region.

In contrast, GFP-Rap2C(A-HVR) is unable to rescue brush border formation in Rap2A depleted cells (figure 4A & B). In agreement with this, localization of GFP-Rap2C is not affected by replacing the hypervariable region, again indicating that the dominant localization signal of Rap2C is outside the HVR (figure 4A).

A striking difference between the HVRs of Rap2A and Rap2B is their differential CAAX box modification. Whereas the CAAX motif of Rap2A results in a farnesylated protein, the Rap2B CAAX motif is modified with a geranylgeranyl moiety [17].

**Figure 5:**
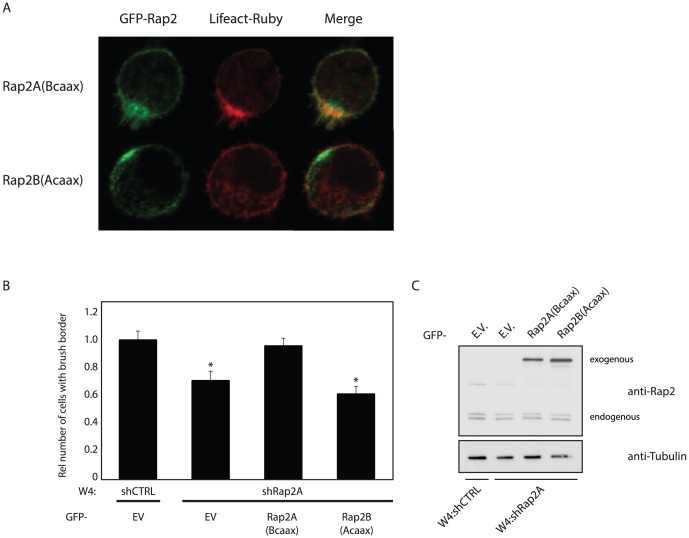
CAAX-box swopped Rap2A and Rap2B mutants function indistinguishable from the wild type Rap2 proteins. A: Images of Rap2A depleted W4 cells expressing GFP-tagged CAAX-swopped Rap2 constructs and Lifeact-Ruby. B: Quantification of brush border formation in Rap2A-depleted W4 cells in which GFP-tagged CAAX-box mutant Rap2A and Rap2B were expressed. (Total counts ∼150 cells per condition) *p<0,05 using paired samples t-test. C: Western blot of lysates from a rescue experiment probed with anti-Rap2 antibody and anti-αTubulin as a loading control. shCTRL: non-targeting control short hairpin, EV: empty vector.

To investigate whether different isoprenyl modifications cause selective activation we generated Rap2A and Rap2B mutants in which CAAX box sequences were interchanged and performed a rescue experiment in Rap2A depleted W4 cells. We find that both geranylgeranylated Rap2A and farnesylated Rap2B are functionally indistinct from wild type Rap2A and Rap2B (figure 5A & B). Therefore, we conclude that differential CAAX modification does not underlie specificity between Rap2A and Rap2B during brush border formation.

## Discussion

Although the Rap2 GTPases are highly similar, they are engaged in isoform specific signaling pathways. For instance, during intestinal polarity establishment isoform specific signaling by Rap2A regulates the formation of the apical brush border. Since the Rap2 proteins are most divergent in their hypervariable region, which affects protein localization, differential localization would be the simplest explanation for isoform specificity. We indeed show that in polarized intestinal epithelial cells Rap2C is localized in cytoplasmic vesicles, whereas Rap2A is at the plasma membrane and apical recycling endosomes. Nevertheless, Rap2B localizes similar to Rap2A implying that other factors than localization must determine specificity between Rap2A and Rap2B.

We show that in contrast to Rap2A, Rap2B is not activated at the apical plasma membrane during brush border formation. Furthermore, a constitutively activated Rap2B(V12) mutant is able to restore brush border formation in Rap2A-depleted cells, indicating that the failure of Rap2B to become activated determines specificity between Rap2A and Rap2B.

We show that differential activation is imposed by the C-terminal hypervariable region, which also determines localization of Rap2A and Rap2B. Importantly, the hypervariable region regulates Rap2 activity independently of its effects on proteins localization as both Rap2A and Rap2B localize similarly in polarized epithelial cells.

Exactly what feature of the HVR allows for selective activation of Rap2A remains unknown. The hypervariable region of Rap2 comprises the C-terminal 14 amino acids of which four residues differ between Rap2A and Rap2B. Importantly, next to these four residues the HVR of Rap2A and Rap2B is differentially isoprenylated as a result of different CAAX motifs: whereas Rap2A is modified with a farnesyl moiety, Rap2B is equipped with a geranylgeranyl moiety. Although differential isoprenylation could allow for isoform specific binding partners [10], we find that differential CAAX modification does not underlie signaling specificity between Rap2A and Rap2B during brush border formation.

Our study thus suggests that subtle differences in het HVR sequence affect the ability of PDZGEF to activate Rap2B. The involvement of the C-terminus in determining specificity of a GEF is not without precedence. For instance, a proline-rich sequence in the carboxy-terminus of Rac1 allows specific binding of the SH3-domain containing GEF β-PIX [18]. In addition, the C-terminus can regulate activity of GTPases by other means than providing a direct binding site for GEFs. For example, a polybasic motif in the hypervariable region of Rac1 allows binding of Extracellular Signal-Regulated Kinase (ERK) that by phosphorylating Rac1 at position T108 affects GEF binding [19]. For Rap2, so far no isoform specific interaction partners have been documented that could explain selective activation of Rap2A. However, our data suggests that the HVRs of Rap2A and Rap2B engage in isoform specific interactions that results in the selective activation of Rap2A.

In conclusion, we identify a function for the hypervariable region of the Rap2 proteins in regulating protein activity independent of its function in regulating protein localization during brush border formation.

## Supporting Information

File S1 Contains the following files: Figure S1: Relative expression levels of Rap2 isoforms in W4 cells infected with pooled shRap2A hairpins or a single (#5) shRap2A hairpin. RNA from W4:shRap2A cells was extracted and subjected to QPCR for the Rap2 isoforms. Average relative expression levels were determined in three QPCR experiments from independent RNA extractions. Error bars indicate standard deviation. *p<0,001. Figure S2: Rap2C activity is not induced during W4 cell polarization. Figure S2: Pulldown of GTP-bound Rap from W4 cells tranfected with V5-Rap2C with or without 20 h of doxycycline stimulation. Figure S3: Constitutively activated Rap2C(V12) can not restore brush border formation in Rap2A depleted W4 cells. Figure S3A: Image of Rap2A-depleted cell expressing GFP-Rap2C(V12) and the actin marker Lifeact-Ruby. Figure S3B: Quantification of brush border formation in W4 cells stably depleted of endogenous Rap2A expressing GFP or GFP-tagged Rap2C(V12) (total counts ∼200 cells per condition). *p<0,05 using paired samples t-test. Figure S4: Rap2A(B-HVR) is less active compared to Rap2B(A-HVR) in polarized W4 cells. GTP-Rap pulldown from W4 cells transfected with V5-Rap2B, V5-Rap2B(A-HVR) or V5-Rap2A(B-HVR) after doxycline-induced polarization. Results from three independent pulldown experiments were quantified and expressed as ratios of GTP-Rap2 vs. total Rap2 relative to Rap2B. Error bars represent the standard deviation in the ratios of independent experiments. Supplementary methods. RNA from W4 cells infected with four different or a single (#5) shRap2A hairpin was isolated using the RNeasy Mini Kit (Qiagen). RNA concentration was quantified and 2 µg of RNA was converted to cDNA using the iScript cDNA synthesis kit (Bio-Rad). cDNA from control infected W4 cells was diluted to generate a reference dilution series and cDNA levels were quantified by SYBR green real-time PCR on a C1000 Thermal Cycler (Bio-Rad). The following primer sequences were used: Rap2A-FW 5′-CATGCTGTTCTGCATGTAAC-3′, Rap2A-RV 5′-CAAGTTCTGCAGTGGAGTAG-3′, Rap2B-FW 5′-GACTGATTGCGATTCTGAGG-3′, Rap2B-RV 5′-CACACTGTATTGGCATCAGT-3′, Rap2C-FW 5′-CAGGATATCAAGCCAATGAG-3′, Rap2C-RV 5′-CTGAAGACATAACCTCTCTTTC-3′. Expression levels were normalized to HPRT1 and GAPDH mRNA levels. Relative expression levels of thee QPCR experiments were averaged and pooled standard deviations were calculated. Average relative expression levels of the two W4:shRap2A samples were compared to the W4:shCTRL reference using independent samples Students' t-test.
(EPS)
